# Comparative Analysis of Podocyte Foot Process Morphology in Three Species by 3D Super-Resolution Microscopy

**DOI:** 10.3389/fmed.2018.00292

**Published:** 2018-10-30

**Authors:** Nadine Artelt, Florian Siegerist, Alina M. Ritter, Olaf Grisk, Rabea Schlüter, Karlhans Endlich, Nicole Endlich

**Affiliations:** ^1^Institute for Anatomy and Cell Biology, University Medicine Greifswald, Greifswald, Germany; ^2^Institute for Physiology, University Medicine Greifswald, Karlsburg, Germany; ^3^Imaging Center of the Department of Biology, University of Greifswald, Greifswald, Germany

**Keywords:** super-resolution microscopy, nephrin, slit diaphragm, podocyte foot process morphology, effacement

## Abstract

Since the size selectivity of the filtration barrier and kidney function are highly dependent on podocyte foot process morphology, visualization of foot processes is important. However, the size of foot processes is below the optical resolution of light microscopy. Therefore, electron microcopy has been indispensable to detect changes in foot process morphology so far, but it is a sophisticated and time-consuming technique. Recently, our group has shown that 3D structured illumination microscopy (3D-SIM), a super-resolution microscopy (SRM) technique, can visualize individual foot processes in human biopsies. Moreover, we have developed a software-based approach to directly quantify the structure of podocyte foot processes named *Podocyte Exact Morphology Measurement Procedure (PEMP)*. As shown in patients suffering from minimal change disease (MCD), PEMP allows the quantification of changes of the foot process morphology by measuring the filtration slit density (FSD). Since rodents are frequently used in basic research, we have applied PEMP to quantify foot processes of mice and rats. Comparative analysis of nephrin-stained kidneys from humans, rats, and mice showed significant differences of the FSD. The highest FSD was measured in mice (3.83 ± 0.37 μm^−1^; mean ± SD) followed by rats (3.36 ± 0.42 μm^−1^) and humans (3.11 ± 0.26 μm^−1^). To demonstrate that PEMP can be used to determine foot process morphology also in affected animals, we measured the FSD in palladin-knockout mice on a 129S1 genetic background compared to wild-type littermates. Taken together, we established a method for the quick and exact quantification of podocyte foot process morphology which can be applied to diagnosis and basic research.

## Introduction

Since the function of the filtration barrier is dependent on podocyte foot process morphology, ultrastructural analyses of kidney biopsies as well as of kidney sections of animal models are important. However, the size of podocyte foot processes and the slit diaphragm in between is below the optical resolution limit of light microscopy and therefore electron microscopy (EM) had to be used for analysis in the past. However, ultrastructural evaluation of the podocyte morphology by EM has some disadvantages: On one hand, the preparation procedure is very sophisticated as well as time consuming and generates a bias due to a dependence on the sectioning angle of the probe (1). On the other hand, the analysis is only subjective and qualitative and represents only a small area of the total kidney section.

Recently, it has been demonstrated that super-resolution microscopy (SRM) overcomes the optical resolution limit of 200 nm formulated by Ernst Abbe. Therefore, different research groups have focused their attention on the visualization of podocyte foot processes by SRM (2, 3). The term SRM summarizes a variety of different techniques like photo-activated localization microscopy (PALM), (direct) stochastic optical reconstruction microscopy [(d)STORM], stimulated emission depletion (STED) and structured illumination microscopy (SIM). SIM is the technique with the lowest optical resolution (85–100 nm in x,y-plane) of all SRM techniques. It allows using standard preparation procedures, normal dyes and mounting media. Furthermore, 3-dimensional structured illumination microscopy (3D-SIM) can be performed with a resolution in the z-plane of 300 nm.

The technique of SIM is based on the generation of Moiré patterns by illuminating the sample through a grid, which is shifted and rotated. Moiré patterns change the spatial frequencies in such a way that the optical resolution is doubled. To generate a SIM picture, three to five different angles and five phase shifts of the grid are necessary resulting in 15–25 widefield (WF) pictures that can be reconstructed by a specific algorithm to extract the high-frequency information into a high-resolution picture (4, 5). We have already visualized and measured individual podocyte foot processes in formalin-fixed and paraffin-embedded (FFPE) human tissue originating from healthy subjects and patients diagnosed for minimal change disease (MCD) by SIM (6). Further, we have established an automatic evaluation of the filtration slit density (FSD), meaning the total length of the slit diaphragm per glomerular capillary area, as a direct parameter to quantify podocyte morphology (6). In combination with classic histology of FFPE sections, the approach named *Podocyte Exact Morphology Measurement Procedure (PEMP)* is able to diagnose MCD along with the quantification of the severity of podocyte injury.

It is well known that foot process morphology is highly dependent on an intact actin cytoskeleton. In this context, mutations in actin-binding proteins that are specifically expressed in podocytes, like α-actinin-4 (7) or anillin (8), induce focal segmental glomerulosclerosis (FSGS). Since the actin-associated protein palladin is highly expressed in podocytes (9), a podocyte-specific knockout mouse was generated (10). In these mice, we found alterations in podocyte morphology as well as an increased susceptibility to develop glomerulopathies compared with wild type mice in response to the injection of nephrotoxic serum (10).

The following study determined the FSD of human, rat and mouse podocytes that were stained for nephrin and the actin-binding protein synaptopodin by 3D-SIM. Furthermore, we quantified the broadening of the podocyte foot processes of palladin-knockout mice (6- and 12-month) backcrossed to a 129S1 background (PodoPalld129^−/−^) compared to control mice by PEMP.

## Materials and methods

### Animal experiments

All studies involving experimental animals were performed in accordance with the German animal protection act and overseen by local authorities of the federal state Mecklenburg-Western Pomerania. Rat kidney tissue from 4-month-old male Wistar rats (Charles River, Sulzfeld, Germany) was used. The animals were kept with humidity (60%) and temperature (22°C) controlled and 12 h day/night cycle. Food and fresh tap water were available *ad libitum*. Rat kidneys were fixed in 4% paraformaldehyde and after dehydration in a graded series in ethanol and clearing in xylene embedded in paraffin. Mice were housed as described previously (11). Podocyte-specific palladin-knockout (PodoPalldBL6^−/−^) mice and controls (PodoPalldBL6^+/+^) with C57BL/6 genetic background were generated as described previously (10) and isolation of glomeruli of 6 month old male mice with magnetic Dynabeads was performed as described before (12). Mice were backcrossed to the 129S1 genetic background and male podocyte-specific palladin-knockout (PodoPalld129^−/−^) and control (PodoPalld129^+/+^) mice were used for 3D-SIM at 6 months and 1 year of age. Genotyping of mice was performed with Phire® Animal Tissue Direct PCR Kit (Finnzymes/Thermo Fisher Scientific, Waltham; MA, USA) in accordance to the manufacturer's instructions using specific primers (10).

### Human biopsies

As healthy controls, anonymized formalin-fixed and paraffin-embedded excess kidney tissue of partial nephrectomies of the Department of Urology of the University Medicine Greifswald was used. The local ethics committee of the University Medicine Greifswald approved the use of the biopsies from Greifswald. All experiments were performed in accordance with local guidelines overseen by the University Medicine Greifswald, Greifswald, Mecklenburg-Western Pomerania.

### 3D-SIM

Sample preparation and imaging was performed as described before (6) with following minor adjustments: 4 μm paraffin sections were directly mounted on #1.5 high precision coverslips (VWR, Radnor, PA, USA) and paraformaldehyde-induced autofluorescence was quenched by incubation for 10 min in 100 mM glycine dissolved in 1x PBS, pH 7.4. Sections were blocked in 1% fetal bovine serum, 1% normal goat serum, 1% bovine serum albumin, and 0.1% cold fish gelatin in PBS for 1 h at room temperature. Primary antibodies diluted in blocking solution were: 1:300 polyclonal guinea pig anti nephrin antiserum (GP-N2), 1:50 monoclonal mouse anti synaptopodin IgG1 antibody (G1D4) (both Progen, Heidelberg, Germany), 1:150 polyclonal rabbit anti synaptopodin IgG (SE-19), 1:150 monoclonal mouse anti α-tubulin IgG1 antibody (T9026) (both Sigma-Aldrich, St. Louis, MO, USA) detected by Cy3-labeled polyclonal donkey anti guinea pig IgG, Alexa 488-labeled goat anti mouse IgG F(ab)_2_ fragment and Cy3-labeled polyclonal goat anti rabbit IgG (H+L) (all from Jackson Immuno Research, Hamburg, Germany) diluted 1:600 in blocking solution. Nuclei were stained for 5 min with DAPI (1:100, Sigma-Aldrich) diluted in PBS. Samples were mounted in Mowiol for microscopy (Carl Roth, Karlsruhe, Germany). WF and 3D-SIM images were acquired on a Zeiss Elyra PS.1 system with five horizontal shifts and five rotations of the illumination pattern with a slice-to-slice distance of 110 nm. 3D-SIM images were reconstructed using Zeiss ZEN black software. To account for chromatic aberration of the optical system, channels were three-dimensionally aligned using values determined by imaging of sub-diffraction fluorescent beads (Tetraspek beads, Molecular Probes, Invitrogen) mounted in the same medium as the biological samples. CLSM micrographs were obtained on a Leica TCS SP5 laser scanning system using a 63x, 1.4 NA oil immersion objective. Digital post-processing, profile plotting and *PEMP* was performed using FIJI (13) combined with the custom-built macro described before (6). Briefly, PEMP was performed on 3D-SIM images of nephrin-stained kidney sections. Areas of the capillary were selected and encircled. The software program determined the total slit diaphragm length (lSD) and the capillary area (A) of the selected region. FSD values were calculated from the ratio of the lSD and A. The following image stacks were analyzed by PEMP: 21–30 glomeruli in three individual rats, 30 glomeruli in four individual humans and 20 glomeruli in three to four individual PodoPalld129^+/+^ and PodoPalld129^−/−^ mice. Student's *t*-test with Bonferroni correction for multiple testing and two-way analysis of variance were used for statistical analysis. Differences were regarded as significant at a *p* < 0.05

### Scanning electron microscopy (SEM)

Glomeruli of 6 month old male PodoPalld^+/+^ and PodoPalld^−/−^ mice were isolated with magnetic Dynabeads as described previously (12). Samples were fixed with 2.5% glutaraldehyde in PBS (pH 7.5) for 1 h at room temperature and then stored at 4°C overnight. After washing with 1x PBS, samples were treated with 1% osmium tetroxide in PBS for 1 h, 1% thiocarbohydrazide for 30 min, and 1% osmium tetroxide in PBS for 1 h at room temperature with washing steps in between. Subsequently, samples were dehydrated in a graded series of aqueous ethanol solutions (10–100%) and then critical point-dried. Finally, samples were mounted on aluminum stubs, sputtered with gold/palladium and examined with a scanning electron microscope EVO LS10 (Carl Zeiss Microscopy GmbH, Oberkochen, Germany). Afterwards, micrographs were edited using Adobe Photoshop CS6.

## Results

### Visualization of podocyte foot processes by widefield microscopy, confocal laser scanning microscopy, and 3D-SIM

**Figure 1:**
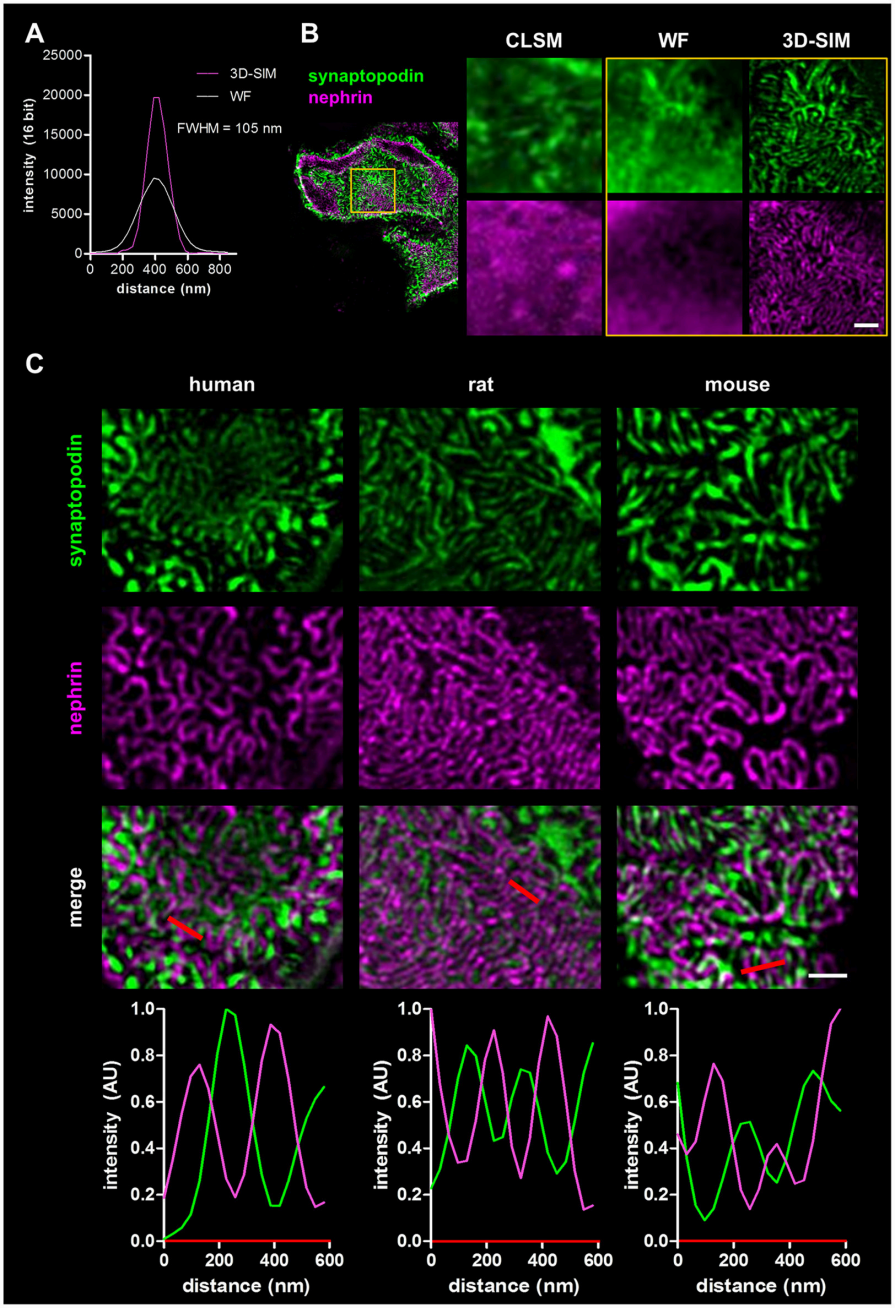
Panel **(A)** shows the resolution of fluorescent beads imaged by wide field (WF) and 3-D structured illumination microscopy (3D-SIM). Panel **(B)** compares the resolution of a healthy rat capillary loop in the glomerulus stained for synaptopodin and nephrin imaged by confocal laser scanning microscopy (CLSM), WF, and 3D-SIM (yellow box). Scale bar represents 1 μm. Panel **(C)** shows synaptopodin and nephrin stained kidney sections of healthy human, rat and mouse. Corresponding intensity profile plots over several foot processes (red lines) are shown at the bottom. Scale bar represents 1 μm.

To demonstrate the increase of the optical resolution by SIM, we imaged immobilized sub-diffraction fluorescent beads by WF and 3D-SIM and measured the full width at the half maximum of the fluorescence intensity (FWHM). As shown in the graph in Figure 1A, our optic setup was able to reach a lateral resolution of ~105 nm.

Four micrometers of FFPE sections of rat kidneys were incubated with antibodies against the slit diaphragm protein nephrin as well as the actin-associated, foot process-specific protein synaptopodin. After staining with the secondary antibodies labeled with Cy3- and Alexa 488, respectively, the kidney sections were visualized by confocal laser scanning microscopy (CLSM), WF microscopy and finally by 3D-SIM. Figure 1B demonstrates that the 3D-SIM images of healthy rat glomeruli show the slit diaphragm (purple) in high resolution as well as the interdigitating foot processes (green) in contrast to images taken by CLSM and WF with a much lower resolution.

Additionally, rat kidney sections were co-stained with an antibody against synaptopodin and the microtubule protein α-tubulin (Supplementary Figure 1, Supplementary Movie 1). 3D-SIM images display the highest resolution of glomerular capillaries surrounded by α-tubulin-positive major processes next to podocyte foot processes stained with synaptopodin.

### Comparative analysis of foot process morphology in humans, rats and mice

In Figure 1C, the corresponding 3D-SIM images of all three healthy species are shown. In humans as well as in rats and mice, the interdigitating synaptopodin-labeled foot processes and the nephrin-positive slit diaphragm can be clearly distinguished. 3D-SIM is therefore able to at least double the optical resolution allowing the study of foot processes and the slit diaphragm in detail.

To compare podocyte foot process morphology of humans, rats and mice, 3D-SIM of stained tissue (nephrin and synaptopodin) was performed. As shown in Figure 1C, human, rat and mouse kidney sections show interdigitating foot processes stained for synaptopodin (green) which are bridged by the slit diaphragm protein nephrin (purple). The individual foot processes of all species were clearly distinguishable from the slit diaphragm. The interdigitating nature of foot processes is also demonstrated by rectangular profile plots over several foot processes demonstrating synaptopodin-fluorescence peak values in areas where no signal for nephrin is detected and vice versa.

**Figure 2:**
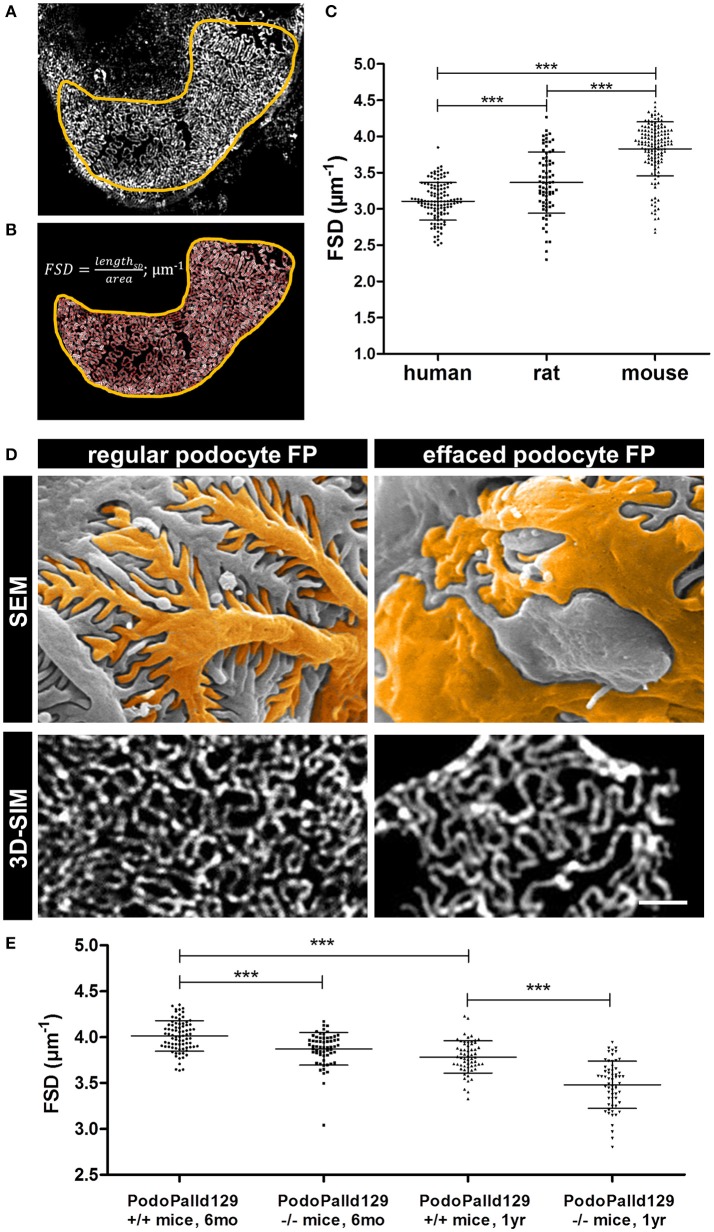
Panel **(A)** shows a murine glomerular capillary area with clearly distinguishable foot processes. Panel **(B)** shows the PEMP principle of automatic segmentation (red) of the slit diaphragm length and quantification of the glomerular capillary area (yellow). The graph in **(C)** shows FSD values of healthy human, rat, and mouse tissue. Horizontal bars indicate mean values; whiskers show the corresponding standard deviations. ^***^p < 0.0005. Panel **(D)** demonstrates regular and effaced podocyte foot processes (FP) of 6 month old control (PodoPalld^+/+^) and podocyte-specific palladin-knockout mice (PodoPalld^−/−^). Upper panels display images taken by scanning electron microscopy (SEM) and were false colored for better visualization. Images below display kidney sections stained for the slit diaphragm protein nephrin imaged by 3D-SIM. Scale bar represents 1 μm. FSD values of 6 month and 1 year old 129S1 control mice (PodoPalld129^+/+^) and 129S1 podocyte-specific palladin-knockout (PodoPalld129^−/−^) mice are shown in **(E)**. Horizontal bars indicate mean values, whiskers show the corresponding standard deviations. ^***^p < 0.001.

Furthermore, we applied our recently developed image processing procedure named PEMP to automatically quantify the changes of the podocyte morphology under different conditions. Using PEMP, the slit diaphragm is automatically segmented (red lines in Figure 2B) in the region of interest (marked by yellow border) and the length of the filtration slit per glomerular capillary area is determined (Figures 2A,B). The ratio of both values is described as the FSD which inversely correlates with the width of podocytes foot processes (d_FP_) (6). The graph in Figure 2C shows the corresponding mean FSD values of the three different species investigated in this study. In human biopsies, we measured a mean FSD of 3.11 ± 0.26 μm^−1^ (mean ± SD, *n* = 121 glomeruli of four humans), whereas rats and mice showed a statistically significant higher FSD of 3.36 ± 0.42 μm^−1^ (*n* = 75 glomeruli of three rats) and 3.83 ± 0.37 μm^−1^ (*n* = 138 glomeruli of six mice), respectively.

### Quantification of podocyte effacement in podocyte-specific palladin-knockout mice on the 129S1 background

Figure 2D shows regular podocytes with well-organized and interdigitating foot processes in scanning electron microscopy (SEM) and a nicely formed slit diaphragm, stained for nephrin, in 3D-SIM. In comparison, injured podocytes with effaced foot processes have broadened podocyte foot processes as shown by SEM and 3D-SIM which visualizes a disorganized pattern of the slit diaphragm.

To check whether PEMP is a method to quantify podocyte injury in animal models, we analyzed 3D-SIM images of podocyte-specific palladin knockout mice on a 129S1 background (PodoPalld129^−/−^ mice). Figure 2E shows the comparison of the mean FSD of PodoPalld129^−/−^ mice at different ages to the corresponding controls (PodoPalld129^+/+^). Genotype and age both significantly affected FSD. We observed that PodoPalld129^−/−^ mice possess a significantly lower FSD (3.87 ± 0.02 μm^−1^; mean ± SEM, *n* = 60 glomeruli of three animals *p* < 0.001) compared to PodoPalld129^+/+^ mice (4.01 ± 0.02 μm^−1^; *n* = 80 glomeruli of four animals) at the age of 6 months. At the age of 12 months, this difference further increased and the FSD for PodoPalld129^−/−^ mice was 3.48 ± 0.03 μm^−1^ (*n* = 60 glomeruli of three animals) in contrast to 3.78 ± 0.02 μm^−1^ (*n* = 60 glomeruli of three animals, *p* < 0.001) in PodoPalld129^+/+^ mice. Interestingly, we also observed a significant difference (*p* < 0.001) between the FSD values in PodoPalld129^+/+^ at the age of 6 vs. 12 month indicating that PEMP can quantify also slight differences of podocyte foot process morphology.

## Discussion

In the past, high resolution visualization of podocyte foot processes as well as of the slit diaphragm spanned between the interdigitating foot processes was only possible by electron microscopy (EM), a very sophisticated and time-consuming technique. Quantification of the foot processes by EM is challenging because this technique allows only the preparation of very thin and small kidney sections. On the other hand, the values obtained by EM highly depend on the sectioning angle of the glomerular capillary. Areas with broadened foot processes, due to sectioning of foot processes along their longitudinal axis, cannot be distinguished from truly effaced areas and lead to false values of foot process morphology as already shown by our group (6). Therefore, the EM results do not correctly represent the real podocyte morphology of biopsies and kidneys taken from patients or animal models, respectively. However, EM analysis remains still valuable for assessing ultrastructural details of podocytes, e.g., the subpodocyte space.

With the development of a new light microscopy technique, the SRM, a new era also for podocyte foot process morphology analysis has started. In contrast to other light microscopy techniques, like CLSM and WF, which allow only a blurred view on foot process morphology and do not reveal any details about the slit diaphragm due to the optical limitation described by Ernst Abbe, a specific type of SRM namely 3D-SIM enabled us to study the course of the slit diaphragm in greater detail. In contrast to STED, another SRM technique allowing an optical resolution down to even 35 nm, 3D-SIM can be directly applied on standard biopsy sections used in pathology routine without any time-consuming optical clearing. Recently, Suleiman and colleagues used Airyscan microscopy to image podocytes of whole murine isolated glomeruli *in situ* stained for synaptopodin (2). As an alternative to SIM, Airyscan has a resolution of 120 nm in the xy-plane and 350 nm in the z-direction, a resolution between that of CLSM and 3D-SIM (14).

Another method to overcome the optical resolution limit was conceived by Grgic and colleagues who imaged individual foot processes in mice carrying an inducible reporter transgene allowing for tamoxifen dose-dependent tomato-expression in podocytes (15). The resolution of CLSM was sufficient to visualize major processes and foot processes, if a labeled podocyte contacted a non-labeled podocyte (15). In that case, the space between single tomato-labeled and non-fluorescent foot processes can be visualized. However, if labeling is to dense, imaging will not work, as only the sub-diffraction and non-fluorescent slit diaphragm (~30 nm) surrounds foot processes. In 2013, Höhne and colleagues overcame this limitation using a similar approach. They modified the mouse model as they used a confetti reporter approach to label neighboring podocytes in different colors (16). In doing so, the researchers showed regularly interdigitating foot processes of adjacent podocytes in isolated murine glomeruli by CLSM. However, this method works only with transgenic animals and cannot be translated to normal mouse, rat, und human kidney tissue.

In the past another SRM technique, (d)STORM, was used to study details of podocyte foot process morphology and podocyte-associated proteins (2). However, only cryosections and specific dyes that can be transferred in a so-called dark state can be used. Further, the quality of the results is highly dependent on the redox buffer system which has to be optimized for each fluorescent dye. Moreover, this technique cannot be used to quantify or to compare morphological changes of foot processes and the slit diaphragm, respectively, in a larger number of animals and biopsies. However, this would be the prerequisite for the pathological routine.

Since some kidney diseases like congenital FSGS are associated with a down regulation of the slit diaphragm protein nephrin (17, 18), alternative slit diaphragm markers like podocin can be used as it was nicely demonstrated by Unnersjö-Jess et al. (3) in a STED-based work on optical cleared kidneys.

The complex podocyte morphology is highly dependent on the actin cytoskeleton and the organization by actin-associated proteins. We have recently shown that palladin, an actin-binding protein (9), plays a pivotal role for the dynamics of the actin cytoskeleton as well as for the stabilization of actin filaments. PEMP analysis of podocyte-specific palladin knockout mice on a specific genetic background (129S1) showed at 6 months as well as at 1 year of age a significant reduction of the FSD compared to the littermates. Moreover, 12-month-old control mice showed significantly more effaced podocyte foot processes compared to mice at 6 months of age, meaning that aging alters podocyte foot process morphology in mice. This is in agreement with the observation of Hassan and colleagues who have already described an age-dependent increase of foot process effacement in podocyte-specific *Sec63/Xbp1* knockout mice in a non-quantitative fashion (19).

Taken together, PEMP is an excellent tool to easily measure podocyte foot process morphology of various species in an exact and quantitative way.

## Ethics statement

This study was carried out in accordance with the recommendations of local guidelines, local ethics committee of the University Medicine Greifswald with written informed consent from all subjects. All subjects gave written informed consent in accordance with the Declaration of Helsinki. The protocol was approved by the University Medicine Greifswald. This study was carried out in accordance with the recommendations of national animal protection guidelines, National Institutes of Health Guide for the Care and Use of Laboratory Animals. The protocol was approved by the local governmental authorities.

## Author contributions

The study was designed by NA, FS, KE, and NE. NA and FS compared the resolution of confocal laser scanning microscopy, wide field, and 3D-SIM. FS conducted comparative analysis of podocyte foot process morphology in humans, rats and mice. NA and AR performed PEMP in PodoPalld129 mice. Scanning electron microscopy was performed by NA and RS. OG provided rat kidney samples. NA, FS, NE, and KE wrote the manuscript. All authors reviewed the manuscript.

### Conflict of interest statement

PEMP (podocyte exact morphology measurement procedures) is registered for a patent. NE, KE, and FS are among the founders of the start-up NIPOKA which will commercialize PEMP. The remaining authors declare that the research was conducted in the absence of any commercial or financial relationships that could be construed as a potential conflict of interest.
