# Supplementary material for: Comparative Analysis of Podocyte Foot Process Morphology in Three Species by 3D Super-Resolution Microscopy
Source: Front Med (Lausanne). 2018 Oct 30;5:292. doi: 10.3389/fmed.2018.00292 (PMC6218959; doi:10.3389/fmed.2018.00292)
Supplement: Supplementary file 2 [file Data_Sheet_1.PDF]

Supplementary Material

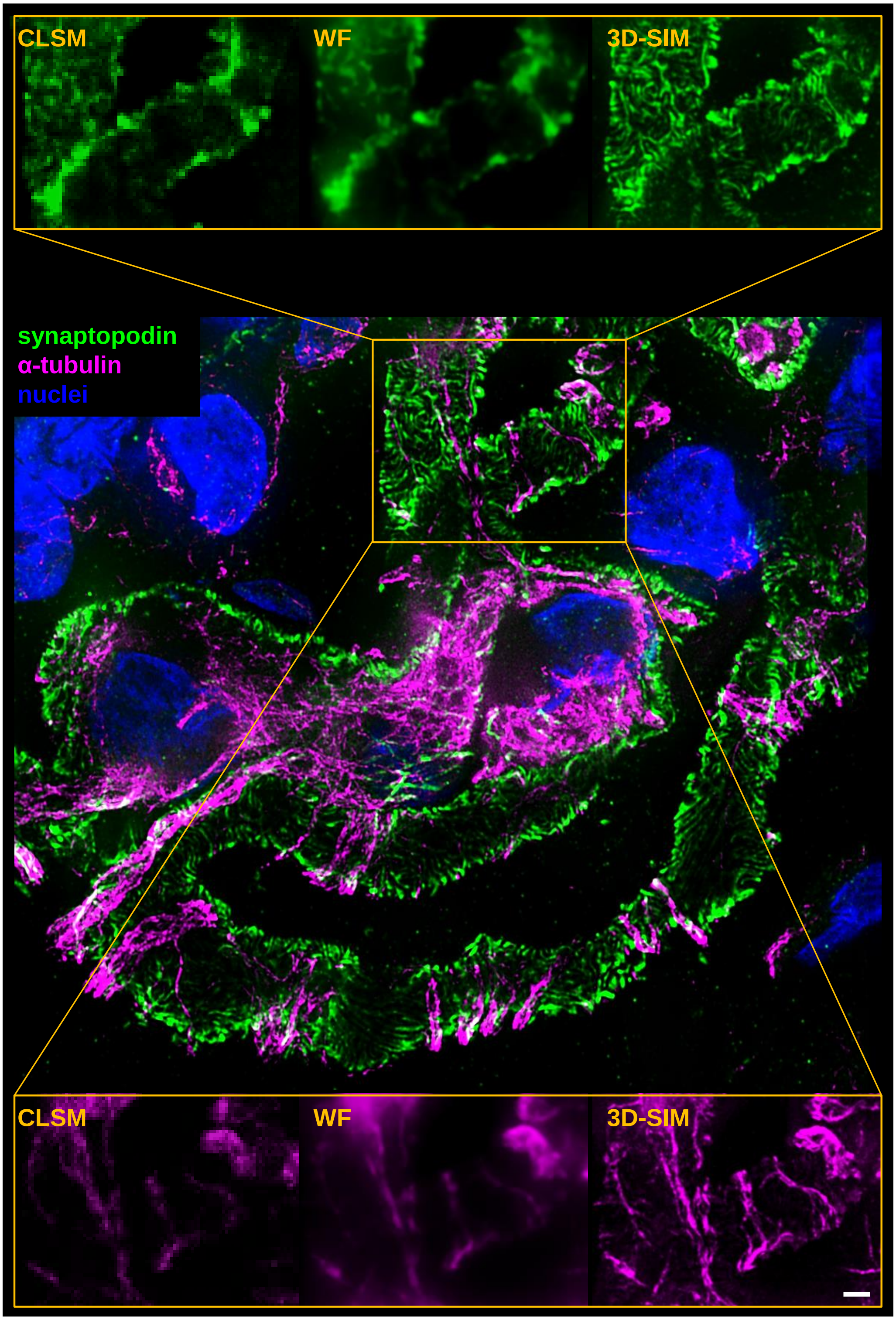

Supplementary Figure 1. Rat kidney section stained for synaptopodin, α-tubulin and nuclei. The resolution of a defined region (yellow box) was compared by CLSM, WF and 3D-SIM. Scale bar represents 1 µm.

Supplementary Movie 1. The movie shows an animated z-stack of a rat kidney section stained for synaptopodin, α-tubulin and nuclei imaged by 3D-SIM.
